# Association between the Suita Score and Body Composition in Japanese Adults: A Large Cross-Sectional Study

**DOI:** 10.3390/nu15224816

**Published:** 2023-11-17

**Authors:** Saori Onishi, Akira Fukuda, Masahiro Matsui, Kosuke Ushiro, Tomohiro Nishikawa, Akira Asai, Soo Ki Kim, Hiroki Nishikawa

**Affiliations:** 1 Second Department of Internal Medicine, Osaka Medical and Pharmaceutical University, Takatsuki 569-8686, Osaka, Japantomohironisikawa5795@yahoo.co.jp (T.N.);; 2 Health Science Clinic, Osaka Medical and Pharmaceutical University, Takatsuki 569-8686, Osaka, Japan; 3 Department of Gastroenterology, Kobe Asahi Hospital, Kobe 653-8501, Hyogo, Japan

**Keywords:** Suita score, body composition, fat mass, fat-free mass, waist circumference

## Abstract

The purpose of this study was to clarify the relationship between the Suita score (a prediction model for the development of cardiovascular disease) and body composition in Japanese health check-up subjects (6873 men and 8685 women). The Suita score includes 8 items (age, gender, smoking, diabetes, blood pressure, low-density lipoprotein, high-density lipoprotein, and chronic kidney disease). Factors associated with the Suita score within body composition-related parameters (body mass index (BMI), waist circumference (WC), fat mass index, fat-free mass index, fat mass to fat-free mass ratio (F-FF ratio), and water mass index) as assessed by bioelectrical impedance analysis were examined. The mean age of subjects was 54.8 years in men and 52.8 years in women (*p* < 0.0001). The mean BMI was 23.9 kg/m^2^ in men and 21.8 kg/m^2^ in women (*p* < 0.0001). Diabetes mellitus was found in 1282 subjects (18.7%) among men and 816 subjects (9.4%) among women (*p* < 0.0001). The mean Suita score was 42.0 in men and 29.6 in women (*p* < 0.0001). In multivariate analysis, WC (*p* < 0.0001), F-FF ratio (*p* < 0.0001), and water mass index (*p* < 0.0001) were independent factors linked to the Suita score for both genders. In conclusion, body composition can be associated with the Suita score in Japanese adults receiving health check-ups.

## 1. Introduction

Because the risk of developing myocardial infarction is extremely low among Japanese people compared to people in the West, the Framingham Risk Score (FRS), a score used in the West to predict the development of cardiovascular disease (CVD) over a 10-year period, is considered inaccurate for Japanese people [1]. The Suita score is a scoring system that predicts the 10-year probability of developing CVD [1]. The risk score was based on 5866 healthy Japanese subjects (2788 men and 3078 women; mean age: 54.5 years) with no history of myocardial infarction or stroke. After an average of 11.8 years of follow-up and observation of 213 cases of CVD incidence, an algorithm was developed to easily predict the 10-year probability of developing CVD by summing the scores assigned to each risk factor [1]. In that study, FRS tended to overestimate the probability of developing CVD. On the other hand, the Suita score can predict the development of CVD almost as well as the actual probability of developing CVD, and the inclusion of chronic kidney disease (CKD), which has received much attention as a risk for coronary artery disease [2,3,4], was found to provide a more accurate prediction [1]. It has also been reported that the Suita score is useful in predicting stroke onset [5].

Body composition refers to fat, muscle, bone, and water [6,7]. Body composition is an important factor as it has been shown to correlate with clinical outcomes in many previous studies [8,9,10,11,12,13]. Sarcopenia, defined by a quantitative and qualitative loss of skeletal muscle, and sarcopenic obesity, which is sarcopenia combined with obesity, are conditions that have received much attention in recent years due to their close relationship to clinical outcomes [14,15,16,17]. New disease concepts such as sarcopenic dysphagia [18], respiratory sarcopenia [19], and osteosarcopenia [20] have also been proposed. However, to our knowledge, there have been no reports examining the relationship between the Suita score and body composition–related factors. The purpose of this study was to clarify the relationship between the Suita score and body composition in Japanese health check-up subjects.

## 2. Patients and Methods

### 2.1. Patients and Body Composition Analysis

Between February 2022 and May 2023, a total of 15,558 consecutive Japanese subjects with data for both the Suita score and body composition–related parameters as assessed by bioelectrical impedance analysis (BIA) were found in our medical records. Since the Suita score is for those 35 years of age and older, those under 35 were excluded [1]. All study subjects received health check-ups at the Osaka Medical and Pharmaceutical University (OMPU) Health Sciences Clinic (OMPU-HSC, an OMPU-attached facility). In the case of subjects taking antihypertensive, hyperlipidemic, and diabetic medications, data at the time of the medical check-up was employed. At the OMPU-HSC, a TANITA body composition analyzer with automatic height rod (DC-270A-N, Tokyo, Japan) was used, and it is a minimally invasive measuring instrument. Measurements were performed in the resting and standing positions after obtaining consent for body composition measurements for all subjects. Fat mass index (F index) was defined as fat mass divided by the square of height (kg/m^2^). Fat-free mass index (FF index) was defined as fat-free mass divided by the square of height (kg/m^2^). F index to FF index ratio (F-FF ratio) was defined as the F index divided by the FF index. Water mass index (W index) was defined as water mass divided by the square of height (kg/m^2^).

### 2.2. The Suita Score

The Suita score is a risk score that predicts the development of CVD in urban residents and includes CKD and other risk factors. The Suita score can more accurately predict the onset of CVDs and is believed to be useful in preventing CVDs [1]. The Suita score was calculated as reported previously [1,21]. The Suita score includes 8 items (age, gender, smoking, diabetes, blood pressure, low-density lipoprotein, high-density lipoprotein (HDL) and CKD as evaluated by estimated glomerular filtration rate (eGFR)). eGFR (mL/min/1.73 m^2^) was calculated for men as 194 × (serum creatinine (mg/dL))^−1.094^ × (age (years)) ^−0.287^, and for women as 194 × (serum creatinine (mg/dL))^−1.094^ × (age (years)) ^−0.287^ × 0.739 [1,21].

After each item is scored, the total score is defined as the Suita score (Table 1). The predicted probability of CVD incidence in 10 years is reported to be <1% for Suita scores ≤ 35, 1% for Suita scores 36–40, 2% for Suita scores 41–45, 3% for Suita scores 46–50, 5% for Suita scores 51–55, 9% for Suita scores 56–60, 14% for Suita scores 61–65, 22% for Suita scores 66–70, and >28% for Suita scores ≥ 71 [1]. The risk of CVD incidence was categorized using the method reported by Nishimura et al. [1]. According to Nishimura’s study, subjects with a 5% or higher predicted risk were classified into the high-risk group. Thus, we defined subjects with Suita scores ≥ 51 as the high-risk group, 36 ≤ Suita scores ≤ 50 as the intermediate-risk group, and Suita scores ≤ 35 as the low-risk group.

### 2.3. Our Study

First, we investigated the relationship between the Suita score and body composition–related parameters (i.e., body mass index (BMI), waist circumference (WC), F index, FF index, F-FF ratio, and W index) in both genders. Next, body composition–related parameters associated with the Suita score were examined using multivariate analysis. We obtained ethical approval for the study from the ethics committee at OMPU Hospital (approval no. 2023-122) and the protocol strictly observed all regulations of the Declaration of Helsinki. Subject consent with regard to the current study was waived due to the retrospective analysis of the study. 

### 2.4. Statistics

In two-group comparisons (continuous variables), the Student’s *t*-test or Mann–Whitney *U*-test was applied, as appropriate. In multiple-group comparisons (continuous variables), the Kruskal–Wallis test or ANOVA was employed, as applicable. In the group comparisons (nominal variables), the chi-squared test was applied. In the multivariate analyses, multivariate regression analysis with multiple predictive variables using the least squares method was applied to select candidate variables. Unless otherwise noted, data were shown as numbers or mean values (±standard deviation (SD)). Considering the total number of cases exceeded 10,000, the significance level was set at *p* = 0.01. JMP 17.0.0 software (SAS Institute, Cary, NC, USA) was employed to perform statistical analyses.

## 3. Results

### 3.1. Baseline Characteristics

**Figure 1:**
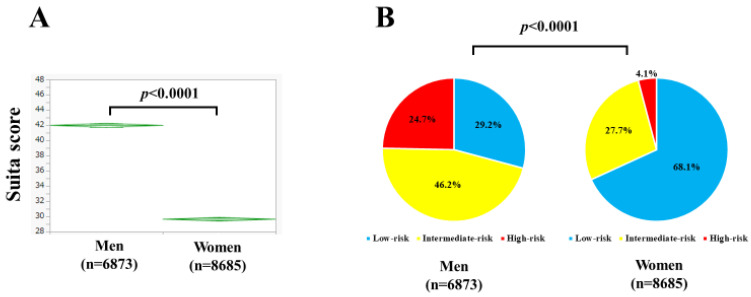
(**A**) The Suita score in men and women. (**B**) The distribution of low-risk, intermediate-risk, and high-risk groups in men and women. The low-risk group was defined as having Suita scores ≤ 35, the intermediate-risk group as 36 ≤ Suita scores ≤ 50, and the high-risk group as Suita scores ≥ 51.

Baseline characteristics in this study (*n* = 6873 for men and *n* = 8685 for women) are shown in Table 2. The mean (±SD) age was 54.8 ± 11.3 years in men and 52.8 ± 10.0 years in women (*p* < 0.0001). The mean (±SD) BMI was 23.9 ± 3.6 kg/m^2^ in men and 21.8 ± 3.7 kg/m^2^ in women (*p* < 0.0001). Diabetes mellitus was found in 1282 subjects (18.7%) among men and 816 subjects (9.4%) among women (*p* < 0.0001). Habitual smoking was found in 1564 subjects (22.8%) among men and 551 subjects (6.3%) among women (*p* < 0.0001). The mean (±SD) Suita score was 42.0 ± 11.5 in men and 29.6 ± 11.5 in women (*p* < 0.0001, Figure 1A). There were 2005 men (29.2%) in the low-risk group, 3172 (46.2%) in the intermediate-risk group, and 1696 (24.7%) in the high-risk group, and 5917 women (68.1%) in the low-risk group, 2410 (27.7%) in the intermediate-risk group, and 358 (4.1%) in the high-risk group (*p* < 0.0001, Figure 1B). 

**Figure 2:**
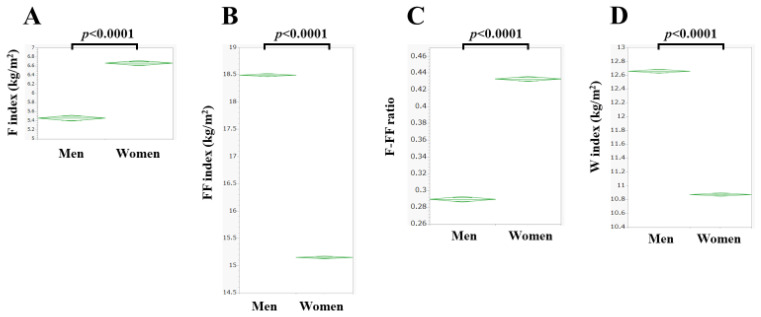
Fat mass index (F index, (**A**)), fat-free mass index (FF index, (**B**)), F-FF ratio (F index divided by FF index, (**C**)), and water mass index (W index, (**D**)) in men and women.

The mean (±SD) F index was 5.46 ± 2.35 kg/m^2^ in men and 6.66 ± 2.92 kg/m^2^ in women (*p* < 0.0001, Figure 2A). The mean (±SD) FF index was 18.49 ± 1.47 kg/m^2^ in men and 15.15 ± 0.98 kg/m^2^ in women (*p* < 0.0001, Figure 2B). The mean (±SD) F-FF ratio was 0.289 ± 0.104 in men and 0.433 ± 0.165 in women (*p* < 0.0001, Figure 2C). The mean (±SD) W index was 12.66 ± 1.34 kg/m^2^ in men and 6.66 ± 2.92 kg/m^2^ in women (*p* < 0.0001, Figure 2D).

### 3.2. The Suita Score According to BMI

**Figure 3:**
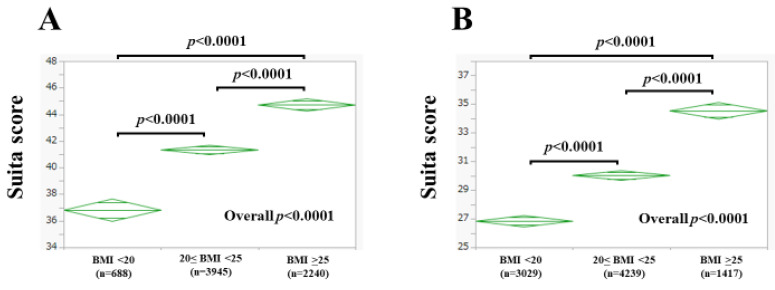
The Suita score according to body mass index (BMI) in men (**A**) and women (**B**).

Among men, there were 688 subjects (10.0%) with BMI < 20 kg/m^2^, 3945 subjects (57.4%) with 20 ≤ BMI < 25 kg/m^2^, and 2240 subjects (32.6%) with BMI ≥ 25 kg/m^2^. The mean (±SD) Suita score was 36.8 ± 11.9 in subjects with BMI < 20 kg/m^2^, 41.3 ± 11.6 in subjects with 20 ≤ BMI < 25 kg/m^2^, and 44.7 ± 10.3 in subjects with BMI ≥ 25 kg/m^2^. The overall difference of the Suita score among the three groups reached significance (BMI < 20 kg/m^2^ vs. 20 ≤ BMI < 25 kg/m^2^: *p* < 0.0001; BMI < 20 kg/m^2^ vs. BMI ≥ 25 kg/m^2^: *p* < 0.0001; 20 ≤ BMI < 25 kg/m^2^ vs. BMI ≥ 25 kg/m^2^: *p* < 0.0001; overall significance: *p* < 0.0001, Figure 3A). 

Among women, there were 3029 subjects (34.9%) with BMI < 20 kg/m^2^, 4239 subjects (48.8%) with 20 ≤ BMI < 25 kg/m^2^, and 1417 subjects (16.3%) with BMI ≥ 25 kg/m^2^. The mean (±SD) Suita score was 26.8 ± 10.7 in subjects with BMI < 20 kg/m^2^, 30.0 ± 11.5 in subjects with 20 ≤ BMI < 25 kg/m^2^, and 34.5 ± 11.0 in subjects with BMI ≥ 25 kg/m^2^. The overall difference of the Suita score among the three groups reached significance (BMI < 20 kg/m^2^ vs. 20 ≤ BMI < 25 kg/m^2^: *p* < 0.0001; BMI < 20 kg/m^2^ vs. BMI ≥ 25 kg/m^2^: *p* < 0.0001; 20 ≤ BMI < 25 kg/m^2^ vs. BMI ≥ 25 kg/m^2^: *p* < 0.0001; overall significance: *p* < 0.0001, Figure 3B). 

### 3.3. The Suita Score According to Waist Circumference

**Figure 4:**
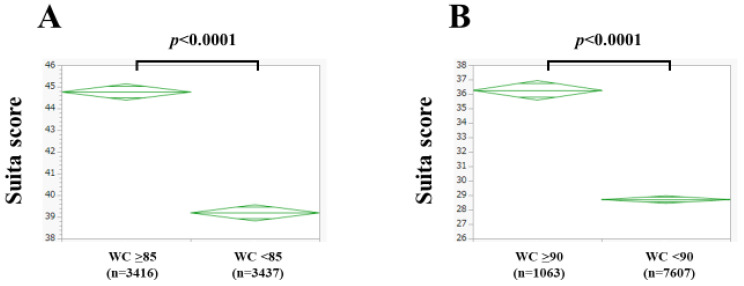
The Suita score according to waist circumference (WC) in men (**A**) and women (**B**).

In Japanese criteria for metabolic syndrome, WC ≥ 85 cm is the cutoff point for men [22]. Among men, there were 3416 subjects (49.8%) with WC ≥ 85 cm, and 3437 subjects (50.2%) with WC < 85 cm (missing data, n = 20). The mean (±SD) Suita score was 44.8 ± 10.7 in subjects with WC ≥ 85 cm, and 39.2 ± 11.6 in subjects with WC < 85 cm (*p* < 0.0001, Figure 4A). 

In Japanese criteria for metabolic syndrome, WC ≥ 90 cm is the cutoff point for women [22]. Among women, there were 1063 subjects (12.3%) with WC ≥ 90 cm, and 7607 subjects (87.7%) with WC < 90 cm (missing data, n = 15). The mean (±SD) Suita score was 36.3 ± 10.8 in subjects with WC ≥ 85 cm, and 28.7 ± 11.2 in subjects with WC < 85 cm (*p* < 0.0001, Figure 4B). 

### 3.4. Body Composition as Assessed by the BIA and Risk Classification of the Suita Score

**Figure 5:**
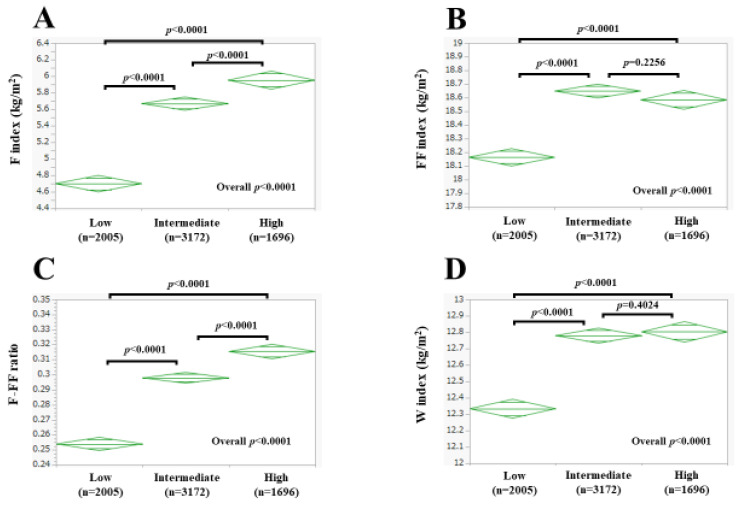
F index (**A**), FF index (**B**), F-FF ratio (**C**), and W index (**D**) according to the risk classification of the Suita score in men. Low indicates low-risk group. Intermediate indicates intermediate-risk group. High indicates high-risk group.

Among men, the mean (±SD) F index was 4.70 ± 1.98 kg/m^2^ in the low-risk group, 5.67 ± 2.46 kg/m^2^ in the intermediate-risk group, and 5.95 ± 2.31 kg/m^2^ in the high-risk group. The overall difference of the F index among the three groups reached significance (low vs. intermediate: *p* < 0.0001; low vs. high: *p* < 0.0001; intermediate vs. high: *p* < 0.0001; overall significance: *p* < 0.0001, Figure 5A). The mean (±SD) FF index was 18.16 ± 1.40 kg/m^2^ in the low-risk group, 18.65 ± 1.52 kg/m^2^ in the intermediate-risk group, and 18.58 ± 1.39 kg/m^2^ in the high-risk group. The overall difference of the FF index among the three groups reached significance (low vs. intermediate: *p* < 0.0001; low vs. high: *p* < 0.0001; intermediate vs. high: *p* = 0.2256; overall significance: *p* < 0.0001, Figure 5B). The mean (±SD) F-FF ratio was 0.254 ± 0.090 in the low-risk group, 0.298 ± 0.107 in the intermediate-risk group, and 0.315 ± 0.103 in the high-risk group. The overall difference of the F-FF ratio among the three groups reached significance (low vs. intermediate: *p* < 0.0001; low vs. high: *p* < 0.0001; intermediate vs. high: *p* < 0.0001; overall significance: *p* < 0.0001, Figure 5C). The mean (±SD) W index was 12.33 ± 1.20 kg/m^2^ in the low-risk group, 12.78 ± 1.39 kg/m^2^ in the intermediate-risk group, and 12.80 ± 1.33 kg/m^2^ in the high-risk group. The overall difference of the W index among the three groups reached significance (low vs. intermediate: *p* < 0.0001; low vs. high: *p* < 0.0001; intermediate vs. high: *p* = 0.4024; overall significance: *p* < 0.0001, Figure 5D).

**Figure 6:**
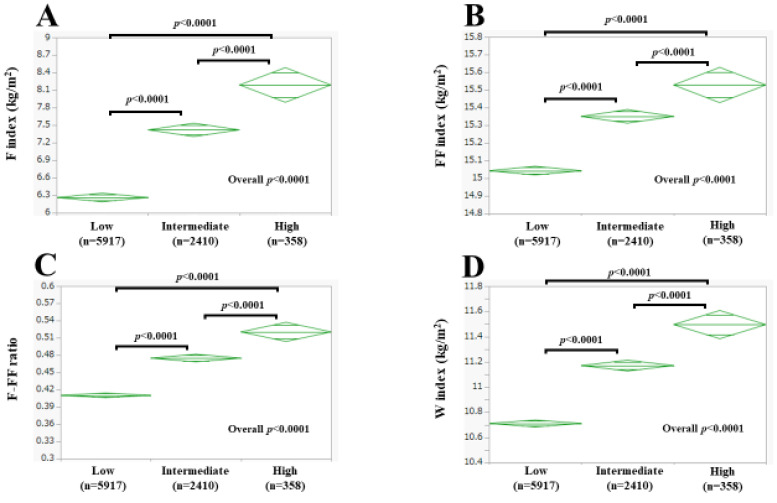
F index (**A**), FF index (**B**), F-FF ratio (**C**), and W index (**D**) according to the risk classification of the Suita score in women. Low indicates low-risk group. Intermediate indicates intermediate-risk group. High indicates high-risk group.

Among women, the mean (±SD) F index was 6.26 ± 2.62 kg/m^2^ in the low-risk group, 7.42 ± 3.33 kg/m^2^ in the intermediate-risk group, and 8.19 ± 3.18 kg/m^2^ in the high-risk group. The overall difference of the F index among the three groups reached significance (low vs. intermediate: *p* < 0.0001; low vs. high: *p* < 0.0001; intermediate vs. high: *p* < 0.0001; overall significance: *p* < 0.0001, Figure 6A). The mean (±SD) FF index was 15.04 ± 0.95 kg/m^2^ in the low-risk group, 15.35 ± 1.02 kg/m^2^ in the intermediate-risk group, and 15.53 ± 0.87 kg/m^2^ in the high-risk group. The overall difference of the FF index among the three groups reached significance (low vs. intermediate: *p* < 0.0001; low vs. high: *p* < 0.0001; intermediate vs. high: *p* < 0.0001; overall significance: *p* < 0.0001, Figure 6B). The mean (±SD) F-FF ratio was 0.410 ± 0.149 in the low-risk group, 0.475 ± 0.185 in the intermediate-risk group, and 0.520 ± 0.180 in the high-risk group. The overall difference of the F-FF ratio among the three groups reached significance (low vs. intermediate: *p* < 0.0001; low vs. high: *p* < 0.0001; intermediate vs. high: *p* < 0.0001; overall significance: *p* < 0.0001, Figure 6C). The mean (±SD) W index was 10.71 ± 1.04 kg/m^2^ in the low-risk group, 11.17 ± 1.18 kg/m^2^ in the intermediate-risk group, and 11.50 ± 1.09 kg/m^2^ in the high-risk group. The overall difference of the W index among the three groups reached significance (low vs. intermediate: *p* < 0.0001; low vs. high: *p* < 0.0001; intermediate vs. high: *p* < 0.0001; overall significance: *p* < 0.0001, Figure 6D).

### 3.5. Multivariate Analyses of Six Body Composition–Related Covariates Linked to the Suita Score

Among men, multivariate analysis of six body composition–related covariates linked to the Suita score (i.e., BMI, WC, F index, FF index, F-FF ratio, and W index) found that WC (*p* < 0.0001), F-FF ratio (*p* < 0.0001), and W index (*p* < 0.0001) were significant factors (set significance level: *p* < 0.01) associated with the Suita score (Table 3A). Likewise, among women, multivariate analysis of the six body composition–related covariates linked to the Suita score found that WC (*p* < 0.0001), F-FF ratio (*p* < 0.0001), and W index (*p* < 0.0001) were significant factors (set significance level: *p* < 0.01) associated with the Suita score (Table 3B).

## 4. Discussion 

In recent years, there has been great interest in accurately assessing the effects of body composition in the fields of nutritional and clinical medical research. This is a significant development in the study of body composition not only in terms of morphology, but also in relation to physiological functions and diseases of the human body. Currently, CVDs based on overnutrition and lack of exercise are increasing worldwide. The Suita score is a model developed in Japan to predict CVD incidence, and the use of the Suita score is recommended in the 2017 Japanese Guidelines for the prevention of atherosclerotic disease. However, although its usefulness has been verified, there are no reports to our knowledge that have examined the relationship between the Suita score and body composition in detail. In these days of increasing academic interest in body composition, we thought it would be clinically significant to clarify this point, which led us to conduct this study. One of the strengths of this study is that it is a large-scale study with a total of over 15,000 cases (a sufficient number of cases for both men and women). 

In the summary of our results, body composition–related factors were closely linked to the Suita score in both men and women. Especially in women, there were significant differences in all comparisons, as shown in Figure 3, Figure 4 and Figure 6. These results indicate that body composition is closely correlated with the presence of CVD. In this study, age, frequency of smoking, blood pressure, presence of diabetes, HDL, and eGFR differed markedly between men and women, resulting in significant differences in the Suita score between genders. On the other hand, in our previous study of patients with metabolic dysfunction–associated fatty liver disease, we reported that the F index, FF index, and F-FF ratio were significantly different between men and women. In the present study, not only the F index, FF index, and F-FF ratio but also the W index differed significantly between men and women [23]. Men have more muscle mass and women have more fat mass. In men, adipose tissue tends to accumulate around the trunk and abdomen, while, in women, adipose tissue usually accumulates around the thighs and buttocks [6]. Body water mass increases with higher BMI (in our current data, BMI significantly correlated with W index for both men (correlation coefficient *r* = 0.73, *p* < 0.0001) and women (correlation coefficient *r* = 0.89, *p* < 0.0001), not shown in the results section) [24]. The significant difference in BMI between men and women may be associated with the significant difference in the W index between genders.

In this study, WC, F-FF ratio, and W index were extracted as independent factors associated with the Suita score for both men and women in multivariate analysis (set significance level, *p* < 0.01). WC is closely related to the accumulation of visceral fat, and visceral fat excess is a risk factor for the development of coronary artery disease [25]. Adipose tissue–derived endocrine factors are collectively conceptualized as adipocytokines, which are the material basis of the pathogenesis of metabolic syndrome [26]. Zhou et al. reported that increased fat-to-muscle ratios were closely linked to an increased risk of CVDs [27], which is in accordance with our current results. A higher fat-to-muscle ratio can be also linked to sarcopenic obesity, which can be more associated with CVDs than sarcopenia or obesity alone [28,29,30]. It seems that body composition is not an individual factor, but a balance among factors. In fact, the F index and FF index were not significant factors in our multivariate analysis. On the other hand, excessive body water is taxing on the heart, which may be why the W index was an independent factor [31]. BMI was not a significant factor linked to the Suita score for either men or women in the multivariate analysis. BMI indices may not adequately capture fat overload, which is a risk for CVD incidence [32].

We should acknowledge several limitations to the current research. First, this study was a cross-sectional single-site observational research using retrospective analysis, and was limited to Japanese adults aged 35 years or older. As mentioned earlier, the Suita score is for people 35 years of age and older. Second, details regarding smoking habits and oral medications were based on individuals’ reports, potentially leading to bias. Thus, the results should be interpreted with sufficient caution and care. Nevertheless, our data indicate that WC, F-FF ratio, and W index were closely linked to the Suita score. The current large number of cases appears to be one strong point in drawing a definite conclusion.

## 5. Conclusions

In conclusion, body composition can be associated with the Suita score, which has been well validated for CVD incidence, in Japanese adults receiving health check-ups. Daily life management with an awareness of body composition may reduce the incidence of CVD. 

## Figures and Tables

**Table 1:** Calculation of the Suita score.

Risk Factors	Category	Points
Age (years)	35–44	30
	45–54	38
	55–64	45
	65–69	51
	70 or older	53
Gender	Men	0
	Women	−7
Current smoker	Yes	5
	No	0
Diabetes	Yes	6
	No	0
Blood pressure	Optimal	−7
	Normal or high normal	0
	Hypertension (stage 1)	4
	Hypertension (stage 2 or higher)	6
LDL (mg/dL)	less than 100	0
	100–139	5
	140–159	7
	160–179	10
	180 or more	11
HDL (mg/dL)	less than 40	0
	40–59	−5
	60 or more	−6
eGFR (mL/min/1.73 m^2^)	more than 60	0
	30–60	3
	less than 30	14

LDL: low-density lipoprotein; HDL: high-density lipoprotein; eGFR: estimated glomerular filtration rate.

**Table 2:** Baseline characteristics.

	Men (n = 6873)	Women (n = 8685)	*p* Value
Age (years)	54.8 ± 11.3	52.8 ± 10.0	<0.0001
Body mass index (kg/m^2^)	23.9 ± 3.6	21.8 ± 3.7	<0.0001
Waist circumference (cm)	85.8 ± 9.7	78.5 ± 9.9	<0.0001
Systolic blood pressure (mmHg)	123.4 ± 16.8	116.4 ± 17.3	<0.0001
Diastolic blood pressure (mmHg)	79.8 ± 12.3	72.6 ± 11.9	<0.0001
Diabetes mellitus, yes/no	1282/5591	816/7869	<0.0001
Fasting blood sugar (mg/dL)	96.7 ± 20.1	89.0 ± 12.9	<0.0001
White blood cell count (/μL)	5622 ± 1520	5090 ± 1354	<0.0001
Hemoglobin (g/dL)	14.9 ± 1.1	13.1 ± 1.1	<0.0001
Platelet count (×10^4^/μL)	24.4 ± 5.4	25.7 ± 6.2	<0.0001
Serum albumin (g/dL)	4.37 ± 0.26	4.30 ± 0.24	<0.0001
AST (IU/L)	24.3 ± 11.6	21.1 ± 8.1	<0.0001
ALT (IU/L)	26.3 ± 18.4	17.6 ± 11.4	<0.0001
ALP (IU/L)	69.0 ± 19.3	64.3 ± 19.9	<0.0001
GGT (IU/L)	45.4 ± 51.3	23.8 ± 24.7	<0.0001
eGFR (mL/min/1.73 m^2^)	70.2 ± 13.4	73.4 ± 13.2	<0.0001
Uric acid (mg/dL)	6.2 ± 1.3	4.6 ± 1.0	<0.0001
HDL (mg/dL)	62.9 ± 16.7	78.5 ± 18.2	<0.0001
LDL (mg/dL)	121.6 ± 30.6	122.2 ± 30.4	0.1994
Habitual smoking, yes/no	1564/5309	551/8134	<0.0001
The Suita score	42.0 ± 11.5	29.6 ± 11.5	<0.0001
Fat mass index (kg/m^2^)	5.46 ± 2.35	6.66 ± 2.92	<0.0001
Fat-free mass index (kg/m^2^)	18.49 ± 1.47	15.15 ± 0.98	<0.0001
F-FF ratio	0.289 ± 0.104	0.433 ± 0.165	<0.0001
Water mass index (kg/m^2^)	12.66 ± 1.34	10.87 ± 1.11	<0.0001

Data are shown as numbers or means (±standard deviation). AST, aspartate aminotransferase; ALT, alanine aminotransferase; ALP, alkaline phosphatase; GGT, γ-glutamyl transpeptidase; eGFR, estimated glomerular filtration rate; HDL, high-density lipoprotein; LDL, low-density lipoprotein; F-FF ratio was defined as F index divided by FF index.

**Table 3:** Multivariate analyses of body composition–related covariates linked to the Suita score in men and women.

(A)			
Men	Estimates	Standard Error	*p* Value
Body mass index	0.0941	4.339	0.9827
Waist circumference	0.6478	0.0345	<0.0001
Fat mass index	−9.660	4.381	0.0275
Fat-free mass index	−6.019	4.355	0.1670
F-FF ratio	204.573	11.852	<0.0001
Water index	6.797	0.260	<0.0001
**(B)**			
**Women**	**Estimates**	**Standard Error**	***p* Value**
Body mass index	−4.312	3.797	0.2562
Waist circumference	0.8616	0.0267	<0.0001
Fat mass index	−0.9486	3.836	0.8047
Fat-free mass index	−9.009	3.834	0.0188
F-FF ratio	43.844	9.076	<0.0001
Water index	14.459	0.493	<0.0001

F-FF ratio was defined as fat mass index divided by fat-free mass index.

## Data Availability

Data available on request due to restrictions eg privacy or ethical.

## Abbreviations

FRS, Framingham Risk Score; CVD, cardiovascular disease; CKD, chronic kidney disease; BIA, bioelectrical impedance analysis; OMPU, Osaka Medical and Pharmaceutical University; OMPU-HSC, Osaka Medical and Pharmaceutical University Health Sciences Clinic; F index, fat index; FF index, fat-free mass index; F-FF ratio, F index to FF index ratio; W index, water mass index; HDL, high-density lipoprotein; eGFR, estimated glomerular filtration rate; BMI, body mass index; WC, waist circumference; SD, standard deviation; low, low-risk group; intermediate, intermediate-risk group; high, high-risk group.
